# Retraction: Rapamycin and Chloroquine: The *In Vitro* and *In Vivo* Effects of Autophagy-Modifying Drugs Show Promising Results in Valosin Containing Protein Multisystem Proteinopathy

**DOI:** 10.1371/journal.pone.0349712

**Published:** 2026-05-20

**Authors:** The PLOS One Editors

After this article [1] was published, concerns were raised regarding results presented in Figs 3 and 5. Specifically:

- Fig 3G and Fig 3H appear to partially overlap.
- Fig 5F VCP Patient 421/07 Chloroquine-Treated TDP-43 panel in [1] appears similar to the originally published Fig 3D Control Myoblasts JC-1 panel in [2]. The Fig 3D Control Myoblasts JC-1 panel in [2] is corrected in [3].

Co-corresponding author VEK stated that Fig 3H is incorrect and 5F VCP Patient 421/07 Chloroquine-Treated TDP-43 panel in [1] is correct. They provided incomplete underlying image data for Figs 2, 3 and 5. Upon editorial assessment, the *PLOS One* Editors noted additional concerns for the underlying image data provided that call into question the reliability of the published results presented in these figures.

In light of the above concerns, the *PLOS One* Editors retract this article.

AN and VEK did not agree with the retraction, stand by the article’s findings, and apologize for the issues with the published article. KJL, CN, and PGY did not respond directly or could not be reached.
